# Astrocytes: targets in obesity

**DOI:** 10.18632/oncotarget.4297

**Published:** 2015-05-27

**Authors:** Yunlei Yang

**Affiliations:** Department of Neuroscience and Physiology, SUNY Upstate Medical University, Syracuse, NY, USA

Obesity and its associated complications impose a huge burden to our society, and constitute a major cause of death. However, the molecular and circuits mechanisms underlying this disorder are largely unknown. At its core, obesity results from an imbalance between energy intake and energy expenditure. Most work has focused on neuronal control of energy homeostasis, while effective treatments are still lacking. An important but poorly understood element is the roles of glial cells in influencing energy states although glial cells overnumber neurons in the mammalian brain. To fully understand the causes of obesity and to develop new effective clinical therapeutics, it is crucial to consider the functions played by glial cells in the control of feeding behaviors.

The hypothalamic arcuate nucleus (ARC), a key brain region that control energy intake and energy expenditure, integrates synaptic inputs that arise within the hypothalamus and from extra-hypothalamic regions so that whole-body energy homeostasis may be achieved. Neurons have been extensively investigated for the control of energy balance in mammals [1, 2]. For example, the orexigenic agouti-related protein (AGRP) neurons localized in the arcuate nucleus play crucial roles in orchestrating food intake [1, 3]. In addition to behaving as interceptive neurons, the AGRP neurons receive synaptic inputs [4] that contribute to the firing rates of AGRP neurons, which are required to evoke food intake. Thus, synaptic strength integrated at AGRP neurons is likely to be critical for the control of energy states. Meanwhile, it has been well demonstrated that glial cells, particularly astrocytes modulate all aspects of neural function, including synapse transmission and synaptic plasticity. For instance, we were the first to demonstrate the contribution of astrocytes to hippocampal long-term potentiation (LTP) [5]. These results suggest that astrocytes may participate in the regulation of food intake by modulating the activities of the orexigenic AGRP neurons. We performed a series of experiments to test this prediction. As expected, in our recent studies [6] we found that chemogenetic selective activation of the astrocytes localized in the dorsomedial hypothalamus (DMH) counter regulated orexigenic ghrelin-evoked hyperphagia, whereas it facilitated anorexigenic leptin-regulated anorexia. In an opposite direction, chemogenetic astrocyte inactivation enhanced ghrelin-evoked feeding, whereas it blunted leptin-induced anorexigenic effects on feeding. We also identified the important role of a classic gliomodulator adenosine which mediated the astrocytic inhibition on ghrelin-evoked feeding by inactivating orexigenic AGRP neurons in the hypothalamic arcuate nucleus via A1 receptor signaling. Consistently, selective deletion of astrocytic leptin signaling also altered ghrelin and leptin-regulated feeding behaviors [7]. These studies suggest that astrocytes influence food intake by modulating the synaptic strength of appetite control circuits and the activities of feeding neurons, filling an important gap in understanding the functional roles of glial cells in regulating energy states.

Based on the above results, we predict that the capability of astrocytic counter regulating effects on food intake may be impaired during high-fat diet feeding, losing of the control of food intake which eventually leads to overeating-induced obesity. Although much work is needed to investigate this prediction and the involved molecular and circuits mechanisms, in near future we plan to develop an approach to selectively manipulate the astrocytes to reverse food intake and body weight in obesity.

Collectively, our recent studies demonstrate the potential for astrocytes to modulate the intercellular and intracellular signaling pathways to rewire the feeding circuits to balance food intake. We are aware that in addition to the release of inhibitory gliomodulators, such as adenosine and GABA, astrocytes can also release excitatory modulators, such as glutamate and D-serine, leading to the complexity of astrocytes in influencing feeding behaviors. We are also aware how astrocytes are activated physiologically to mediate such effects on food intake and how to measure the astrocyte activity *in vivo* under different conditions, such as energy surfeit and deficit respectively. It would vastly improve our knowledge of understanding and treating obesity by developing an approach to specifically manipulate astrocytic signaling, establishing a more defined “astrocytic code” to counter regulate energy surfeit in obesity.
